# Revisiting the Dependence of Electrical Resistivity on Cu-Rich Precipitates in an Aged Fe-Cu Model Alloy: A Microstructure-Based Prediction Model

**DOI:** 10.3390/ma18040752

**Published:** 2025-02-08

**Authors:** Shengjun Xia, Menglin Gao, Xing Hu, Chunfa Huang, Shuaiheng Liang, Wenlu Zhang, Qiulin Li

**Affiliations:** 1 Shenzhen International Graduate School, Tsinghua University, Shenzhen 518055, China; 2 School of Materials Science and Engineering, Tsinghua University, Beijing 100084, China

**Keywords:** Cu-rich precipitates, RPV embrittlement, electrical resistivity, prediction model, non-destructive evaluation

## Abstract

Nanoscale Cu-rich precipitates (CRPs) play a crucial role in the irradiation embrittlement of reactor pressure vessels (RPVs), and binary Fe-Cu alloys serve as practical models to study the evolution of these precipitates. This study investigates the electrical resistivity of an Fe-1.17 wt.% Cu model alloy aged at 450 °C to enhance the understanding of electrical measurements for the non-destructive assessment of RPV irradiation embrittlement. Multi-level characterization methods were used to obtain quantitative data on multi-scale microstructures, including precipitates, dislocations, and grains. The formation and growth of CRPs were found to align closely with the Johnson–Mehl–Avrami model, and the variation in electrical resistivity showed a strong correlation with the evolution of the microstructure. Combined with detailed quantitative microstructure evolution analysis, an electrical resistivity prediction model that considers microstructural mechanisms has been developed. This model can accurately show the effect of CRPs on resistivity and can potentially be extended to RPV steels with other solute-rich precipitates, with a maximum absolute percentage error not exceeding 5%. These results provide a robust basis for the non-destructive and in-service evaluation of RPV irradiation embrittlement using electrical resistivity.

## 1. Introduction

The reactor pressure vessel (RPV) is an irreplaceable component in a nuclear power plant; it contains the reactor core and coolant and prevents the leakage of radioactive materials [1,2]. However, for fission reactors, critical components lack protection from W-coating [3], and the hardening and embrittlement of RPV materials (usually low-alloy steels) induced by neutron irradiation is a critical degradation phenomenon that limits the long-term safe operation of nuclear reactors. Nanoscale solute-rich precipitates or clusters, along with matrix defects such as dislocation loops, are microstructural factors that are responsible for the deterioration of RPV steels [4,5,6,7]. Specifically, for the Cu-bearing RPV steels used in early nuclear power plants, Cu-rich precipitates (CRPs) contribute the most to the hardening [8,9]. Therefore, investigating the precipitation of Cu is essential for fully understanding the mechanism of irradiation embrittlement. Due to the complex composition and microstructure of RPV steels, Fe-Cu binary alloys are often employed as model materials to simulate the precipitation of CRPs in RPV steels [10,11,12,13,14,15,16,17,18]. Ishino et al. found that the electrical resistivity of Fe-Cu alloys decreased with increasing irradiation dose under electron and ion irradiation, which was attributed to Cu precipitation [13]. Tobita et al. reported that the variation in electrical resistivity closely correlated with irradiation hardening associated with Cu precipitation [14]. These studies are very instructive for developing in situ detection methods for irradiation embrittlement, as electrical resistivity testing requires no complex sample preparation and can be evolved into non-destructive testing methods [19,20,21]. By using microstructure as a medium, it is possible to communicate electrical resistivity and mechanical properties and thus evaluate the degree of microstructure evolution and performance degradation of RPV steels through electrical resistivity. It is necessary to clarify the influence of microstructure evolution on electrical resistivity and establish a predictive model from microstructure to resistivity before using electrical resistivity to evaluate the degradation of RPV steels. However, existing studies focus on the relationship between resistivity and hardness or irradiation dose. The lack of correlation between microstructural parameters and electrical resistivity limits the application of resistivity in this regard.

According to the empirical rule summarized by Norbury [22], the relationship between the Cu content and the electrical resistivity of the alloy can be expressed as the following empirical equation:

(1) $$\rho (\mu \Omega \cdot m)=\rho_{0}+0.04[\mathrm{Cu}]$$

Here, *ρ* and *ρ*₀ represent the resistivity values of the alloy and pure iron at room temperature, while [Cu] represents the weight percent of Cu atoms. Based on Equation (1), a decrease of 0.04 μΩ·m in electrical resistivity corresponds to the precipitation of one weight percent of Cu. Some studies have linked the evolution of CRPs to the variation in electrical resistivity using Equation (1) to investigate the precipitation kinetics of Cu [23,24,25]. Although this empirical equation can effectively describe the variation in electrical resistivity with the Cu precipitation process, it does not consider the distribution characteristics of CRPs, such as average size and number density. Using these microstructure distribution data to predict changes in mechanical properties is very mature, but the correlation between them and electrical resistivity is still unclear, which limits the further use of electrical resistivity for studying the CRP evolution process and evaluating the performance degradation of RPV steels. Moreover, since this equation does not account for the increased electrical resistivity caused by electron scattering from dense, fine precipitates, it may overestimate the amount of Cu precipitation [14] and fail to explain the observed increase in resistivity [26]. Few studies have considered the effect of microstructure distribution characteristics on resistivity changes in order to propose a universal relationship.

From the perspective of reducing the handling difficulty, thermal aging is often used to substitute for neutron irradiation [15,16,17,18]. This study seeks to investigate the quantitative correlation between microstructural evolution and resistivity variation in an aged Fe-Cu RPV model alloy. In addition to the precipitation of CRPs, the evolution of grains and dislocations was also quantitatively analyzed using different characterization methods. The mechanism of microstructure evolution and the property variation in the Fe-Cu alloy during aging was also analyzed. On this basis, the present study constructed a microstructure-based electrical resistivity prediction model that considered the contribution of multi-level microstructure, especially the CRPs. This work provides a robust basis for the in-service evaluation of the irradiation embrittlement of RPV steels using electrical resistivity.

## 2. Materials and Methods

**Figure 1:**
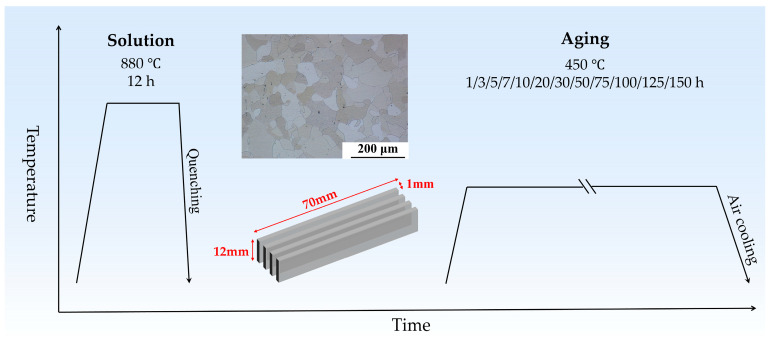
Schematic diagram of the heat treatment process in this study.

The binary Fe-1.2 wt.% Cu alloy used in the present study was supplied by the China Central Iron & Steel Research Institute (Beijing, China). The precise Cu content was found to be 1.17 wt.%, which was determined through inductively coupled plasma analysis. The chemical composition is shown in Table 1. The as-received billet underwent solution treatment at 880 °C for 12 h, followed by water quenching to achieve a uniform initial structure. After the solution treatment, the alloy exhibited a single-phase structure, as depicted in Figure 1.

The solution-treated alloy was cut into small rectangular sheets of 70 × 12 × 1 mm^3^ for the following heat treatment. To accelerate the precipitation of Cu atoms, it is generally necessary to keep the isothermal aging temperature at 500 °C [27]. Aging at lower temperatures allows us to observe the early stages of precipitate formation and the fine-scale microstructure evolution. Referring to our previous research [28], the sheets were aged at 450 °C for different durations, then air cooled to room temperature. The heat treatment process in this study is shown in Figure 1. At least four parallel samples were prepared for subsequent testing and characterization for each processing duration. Vickers hardness tests were performed on the mechanically polished surface of specimens using a Vickers microhardness tester (Mega Instruments, Suzhou, China, AutoVicker 1000AF) with a load of 300 gf. The hardness value for each specimen was obtained by averaging the repeated measurements taken from ten different points on the surface.

The grain distribution and dislocation density of the Fe-Cu alloy samples were characterized by electron backscatter diffraction (EBSD) and x-ray diffraction (XRD) techniques, respectively. The EBSD method can accurately provide statistical information on hundreds of grains in a sample [29], and the XRD method can overcome the errors caused by phenomena such as invisibility and entanglement through direct observation [30]. The EBSD study of the samples was carried out on a scanning electron microscope (Zeiss, Oberkochen, Germany, EVO MA 10) equipped with an EBSD detector (Bruker, Billerica, MA, USA, eFlash FS) using a scanning voltage of 20 kV with a step size of 2 μm. The XRD analysis of the samples was carried out on an x-ray diffractometer (Rigaku, Tokyo, Japan, SmartLab) operated at 45 kV and 200 mA. Each specimen was scanned using Cu Kα radiation from 2θ = 40° to 120° with a scanning speed of 5 °/min and a step size of 0.01°. Each diffraction peak was fitted with the Pseudo-Voigt function to filter out the portion corresponding to Kα2 radiation for subsequent analysis. The EBSD and XRD specimens were first mechanically polished using SiC sandpapers and electrolytically polished using a solution composed of 90% ethanol and 10% perchloric acid at −20 °C under a voltage of 30 V.

The morphology and distribution of the CRPs in the solution-treated and aged samples were analyzed using a field emission transmission electron microscope (JEOL, Tokyo, Japan, JEM-3200FS) using scanning transmission electron microscopy (STEM) mode. TEM specimens, with a diameter of 3 mm, were initially mechanically thinned to a thickness of 50 to 60 μm using SiC sandpapers. Further thinning was then carried out through electropolishing in a twin-jet electropolisher (Smart Innovate, Beijing, China, RL-2) at −20 °C and a voltage of 16 V, until small holes formed in the samples. The electrolytic polishing solution was composed of 6% perchloric acid and 94% ethanol.

Electrical resistivity tests were conducted at 25 °C using a resistance meter (Hioki, Nagano, Japan, RM3545A-1) equipped with a four-point probe, according to the requirements of GB/T 351-2019 [31] and ASTM B193-16 [32]. Three parallel samples were measured for each treatment condition, and each parallel sample was measured three times. The final resistivity value was taken as the average of the nine repeated measurements. Before measuring the electrical resistivity, each sample was polished with SiC sandpapers to remove the surface oxide scale and wire-cutting marks.

## 3. Results

### 3.1. Hardening Behavior

**Figure 2:**
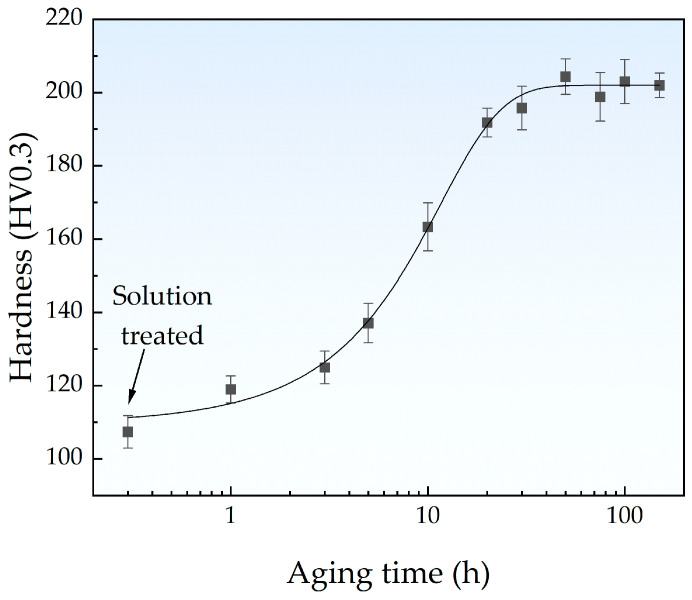
Variation in Vickers hardness for the Fe-Cu alloy samples with aging time.

Figure 2 illustrates the Vickers hardness for the Fe-Cu alloy samples, showing a typical hardening trend throughout the aging treatment. The hardness reached a peak value of 204.3 HV after aging for 50 h and then gradually decreased. This phenomenon is consistent with the findings of Li et al. [25] on an Fe-Cu alloy and Jung et al. [24] on a Cu-bearing medium carbon steel, which can be attributed to the precipitation strengthening effect of CRPs. Based on the hardness variation curve, the solution-treated samples aged for 5 h, 10 h, 50 h, 100 h, and 150 h were selected for further characterization and testing.

### 3.2. Evolution of Dislocations and Grains

**Figure 3:**
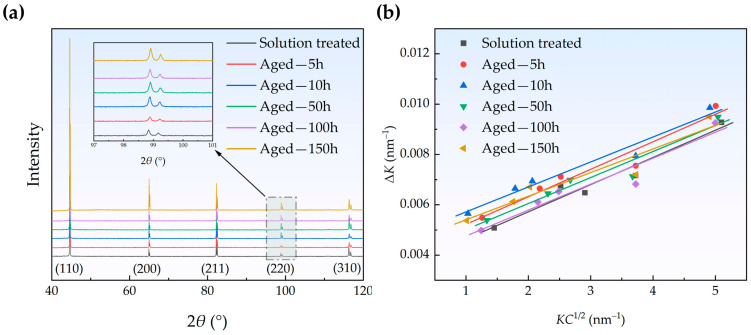
The XRD patterns (**a**) and modified Williamson–Hall plots (**b**) of the solution-treated and aged samples. The dislocation density was calculated from the slope.

The XRD patterns of the samples in different states are shown in Figure 3a. The Miller indices of each diffraction peak are labeled in Figure 3a, and it is evident that the Fe-Cu alloy maintained a single-phase α-Fe structure throughout the aging process. The peak broadening of X-rays attributed to strain has long been investigated in connection with dislocation density [33]. Williamson and Hall [34] proposed a series of equations for calculating dislocation density using XRD peak width, known as the Williamson–Hall (WH) method. This method separates the broadening of diffraction peaks into two contributions—one due to the finite size of the crystallites (size broadening) and the other due to the lattice strain caused by dislocations (strain broadening). This method is beneficial for characterizing the evolution of dislocation density in Fe-Cu alloys during aging since the contributions from size broadening can be ignored for coarse-grained materials [30]. Ungar et al. [35,36] further considered the peak width anisotropy caused by dislocations and derived the modified Williamson–Hall (MWH) method. The MWH method was employed in this study to calculate the dislocation density in the Fe-Cu alloy. The relevant equations are as follows [35,36]:

(2) $$\Delta K=\frac{0.9}{D}+{\left(\frac{\pi M^{2}b^{2}N}{2}\right)}^{\frac{1}{2}}{KC}^{\frac{1}{2}}+O(K^{2}C)$$

(3) $$K=\frac{2\sin\theta }{\lambda }$$

(4) $$\Delta K=\frac{2\cos\theta \Delta \theta }{\lambda }$$

where *θ*, Δ*θ*, and *λ* are the diffraction angle, full width at half maximum (FWHM), and the X-ray wavelength, respectively. *D*, *b*, and *N* are the crystalline size, the magnitude of the Burgers vector, and the dislocation density, respectively. In this work, *b* = 0.25 nm [37]. *M* is a constant parameter that depends on the effective external cutoff radius of dislocations, while *O* represents a negligible higher-order term. According to the research of Wikens [38], *M* = 1 is suitable for non-deformed materials with a low dislocation density; this value is used in this work. *C* is the average contrast factor of dislocations, which is calculated using the following equation for a specific (*h k l*) reflection in the cubic crystal:

(5) $$C=C_{h00}[1+q(\frac{h^{2}k^{2}+h^{2}l^{2}+k^{2}l^{2}}{h^{2}+k^{2}+l^{2}})]$$

where *C_h_*_00_ represents the average contrast factor corresponding to (*h*00) reflection. In this work, *C_h_*_00_ = 0.285 for all samples according to the theoretical calculation in [36]. The *q* parameter is related to the dislocation character and changes between different samples. Since *q* is undetermined, a recursive iteration algorithm based on the least square method was employed to calculate *KC*^1/2^ in the present analysis, and the detailed process can be found in the work of Takebayashi et al. [33].

Figure 3b shows the modified Williamson–Hall plots of different samples, and the dislocation density was calculated from the slope. The results of the evaluation for dislocation density are listed in Table 2. As the aging time increases, the dislocation density of the Fe-Cu alloy decreases slowly from ~1.19 × 10^13^ m^−2^ to ~0.90 × 10^13^ m^−2^. The solution-treated sample has a relatively low dislocation density, consistent with the measurements reported by Liu et al. for an Fe-0.3wt.% Cu alloy [30]. Yin et al. annealed a warm-rolled low-carbon steel at 450 °C for 100 h, and the dislocation density was reduced from 1.06 × 10^14^ m^−2^ to 0.22 × 10^14^ m^−2^ [39]. A similar phenomenon of a significant reduction in dislocation density was also observed in the study of Liu et al. on a deformed SA508Gr.3 RPV steel aged at 450 °C for 2 h [40]. The reduction in dislocation density is mainly due to the annihilation with each other or the sinking in grain boundaries under thermal activation [41]. The precipitation of CRPs hindered dislocation motion, making it more difficult for dislocations to annihilate with each other or sink in grain boundaries.

**Figure 4:**
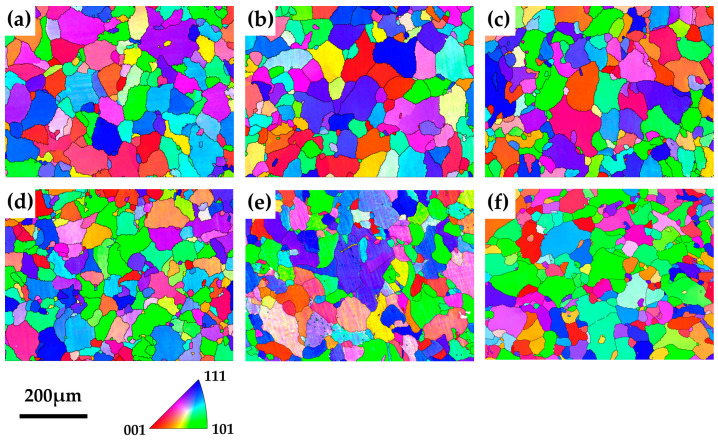
EBSD inverse pole figures (IPFs) of the solution-treated (**a**) and aged for 5 h (**b**), 10 h (**c**), 50 h (**d**), 100 h (**e**), and 150 h (**f**) samples.

Figure 4 shows the inverse pole figures (IPFs) of the solution-treated and aged samples. The distinct colors in Figure 4 indicate different crystal orientations, and the black lines represent high-angle (misorientation > 10°) grain boundaries. The grain orientations of solution-treated and aged samples shown in Figure 4 are random, and no significant orientation change was observed with increasing aging time. The grain morphology shows that the alloy has completely recrystallized and is in the grain growth stage after solution treatment [42]. The statistics for the equivalent circular diameters of grains for solution-treated and aged samples are summarized in Table 2. The average equivalent circular diameters of grains for all samples are in the range of 30–40 μm. The samples’ grain size remained unchanged during aging, with no significant grain growth observed. The precipitation of CRPs pinned the grain boundaries at 450 °C, preventing grain growth [43].

### 3.3. Observation of Cu-Rich Precipitates

**Figure 5:**
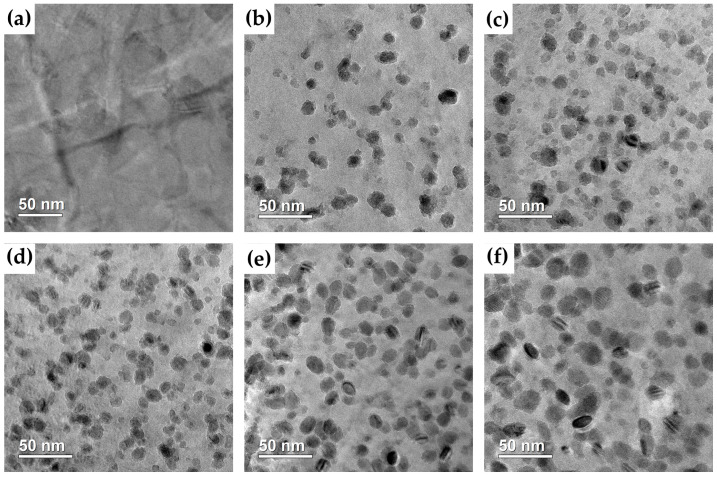
The TEM bright-field images of the solution-treated (**a**) samples aged for 5 h (**b**), 10 h (**c**), 50 h (**d**), 100 h (**e**), and 150 h (**f**).

**Figure 6:**
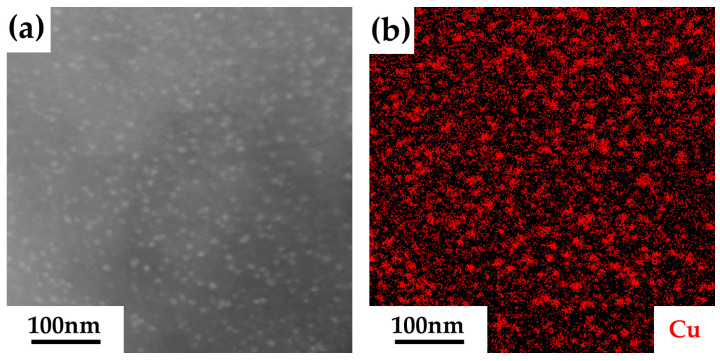
The high-angle annular dark field (HAADF) image (**a**) and energy-dispersive X-ray spectroscopy image (**b**) of the precipitates in the sample aged for 100 h.

Figure 5 presents the TEM bright-field images of the samples in the solution-treated and aged conditions. In Figure 5a, the image of the solution-treated sample shows no evidence of precipitates. However, after aging at 450 °C, numerous nanoscale dark precipitates formed in the α-Fe matrix, as shown in Figure 5b–f. These precipitates were generally evenly distributed, though some agglomeration was also observed. The size of the precipitates increased with prolonged aging time. The elemental analysis image and the high-angle annular dark field (HAADF) image, presented in Figure 6a,b, confirm that the nanoscale precipitates are enriched in Cu.

**Figure 7:**
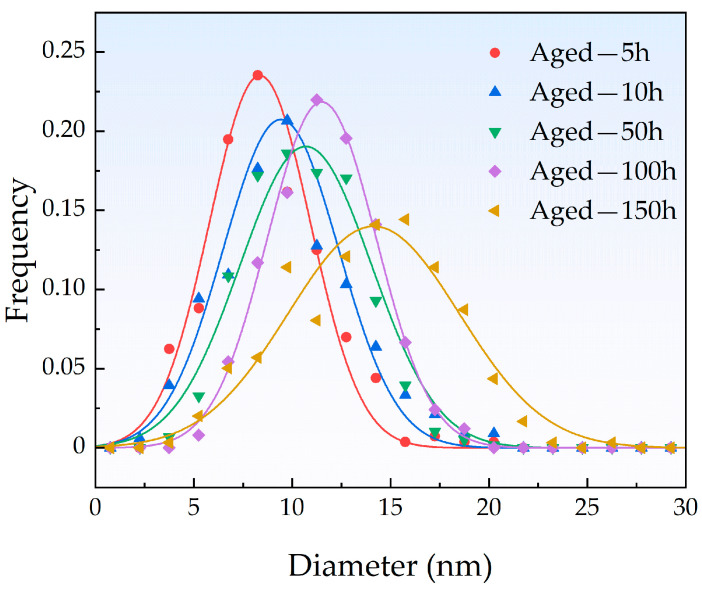
Size distributions of the CRPs in aged samples.

The number density and mean radius of the CRPs in this study were statistically analyzed based on TEM observations, and the volume fraction of the CRPs was then calculated using the mean radius and number density. The statistical results for the size distribution of the CRPs in samples aged for different times are shown in Figure 7 and summarized in Table 3. Both experimental [44,45] and theoretical [16] studies have demonstrated that CRPs initially have a bcc structure, which transitions through a complicated sequence of bcc → 9R → 3R → fcc with size increase. According to previous studies [24,45], the critical sizes for the structure transitions of CRPs from bcc to 9R and from 9R to 3R were determined to be ~5 nm and ~20 nm, respectively. Because the radius of the Cu atom is larger than that of the Fe atom, this transition is driven by the need to reduce strain energy and free energy, with the final fcc precipitates adopting a Kurdjumov–Sachs orientation with the α-Fe matrix. [46]. Specifically, Ahlawat et al. observed a morphological transformation of Cu precipitates from spherical to ellipsoidal shapes, accompanied by a notable change in the aspect ratio [15]. This transformation was linked to the structural transition of the precipitates from 9R to 3R. Therefore, the CRPs observed using TEM in this work should mainly be the 9R structure. The size distribution peak remained relatively narrow up to 100 h, indicating a uniform distribution of CRP sizes. Moreover, the significant increase in the peak width of the size distribution at 150 h indicated that the CRPs had entered the coarsening or Ostwald ripening stage [25,47]. The system sought to minimize the overall energy by redistributing atoms from the smaller precipitates to the larger ones, which decreased the total number of precipitates over time.

## 4. Discussion

### 4.1. Strengthening Mechanism of Cu-Rich Precipitates

The hardening of the Fe-Cu alloy caused by the precipitation of CRPs can be explained by the modulus strengthening mechanism [37]. In a material with heterogeneous microstructures such as a matrix and precipitates, if the precipitates have a different modulus compared to the matrix, the dislocations in the matrix experience an additional resistance when they interact with the precipitates. The difference in shear modulus between the CRPs and the α-Fe matrix creates localized stress concentrations that impede dislocation motion, which enhances the hardness of the Fe-Cu alloy. Based on the model proposed by Russell and Brown, the hardness contribution of CRPs through modulus strengthening can be expressed as follows [37,48]:

(6) $$Hv_{\mathrm{CRP}}=0.58\frac{G_{M}b}{r}{\left[1-{\left(\frac{G_{\mathrm{CRP}}}{G_{M}}\frac{\ln(r/r_{0})}{\ln(R/r_{0})}+\frac{\ln(R/r)}{\ln(R/r_{0})}\right)}^{2}\right]}^{\frac{3}{4}}V_{f}^{\frac{1}{2}}$$

where *G*_M_ and *G*_CRP_ are the shear modulus of the α-Fe matrix and the CRPs, respectively. *G*_M_ = 83 GPa, and the ratio *G*_CRP_/*G*_M_ was estimated to be 0.94 according to the previous study [48]. *b* is determined to be 0.25 nm, while *r*_0_ = 2.5*b* and *R* = 2500*b* are the inner cutoff radius and outer cutoff radius for a dislocation, respectively [37]. *r* is the mean radius of the CRPs and *V*_f_ is the volume fraction of the CRPs. According to Equation (6), the precipitation hardening is strongly influenced by the mean radius *r* and volume fraction *V*_f_ of the CRPs. During the early stages of aging, the volume fraction of CRPs increased rapidly, leading to a continuous hardening of the Fe-Cu alloy. As Cu atom precipitation approached saturation, the increase in volume fraction *V*_f_ slowed down until it remained almost unchanged, and the hardness reached the highest value. A decrease in hardness when aging for a longer duration is associated with the increase in r accompanied by the growth and coarsening of the CRPs [49]. Meanwhile, studies have shown that CRPs with a bcc structure and the same volume fraction have a stronger pinning ability for dislocations than CRPs with a 9R or fcc structure [24,46]. These factors caused a slow decrease in hardness after the peak value.

### 4.2. Kinetics of Cu Precipitation

**Figure 8:**
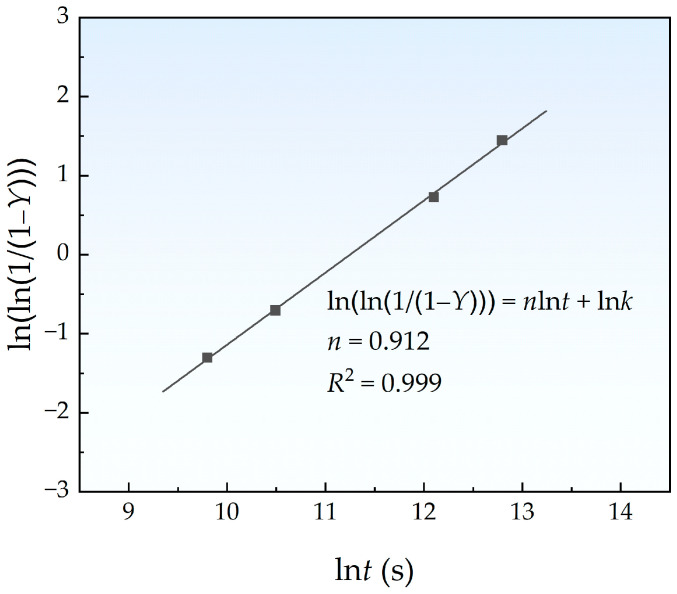
Logarithmic plot of Cu precipitation kinetics at 450 °C.

Table 3 lists the mean radius, number density, and volume fraction of the CRPs in aged samples. The volume fraction of the CRPs peaked after 100 h of aging, close to the value at 50 h, so it can be inferred that the CRPs began to coarsen between 50 h and 100 h. The isothermal phase transformations in metallic systems are formulated by the Johnson–Mehl–Avrami (JMA) theory [50]. The relationship between the transformed fraction *Y* and time *t* can be expressed in the form of a kinetic equation [51]:

(7) $$Y=1-\exp(-kt^{n})$$

where, in this work, *Y* can be calculated from the volume fraction *V*_f_ via $Y=\;V_{f}(t)/V_{f}(150\;h)$. *k* is the rate constant that varies with temperature and *n* is a temperature-independent constant depending on nucleation modes. The lnln(1/(1 − *Y*)) vs. ln*t* plots are shown in Figure 8, and the *R*^2^ of linear fitting was 0.999. The value of *n* calculated from the slope was 0.912, which is in good agreement with those results (*n* < 1) obtained from the experimental [10] and Monte Calo simulation [11] studies.

### 4.3. Electrical Resistivity Prediction Model

**Figure 9:**
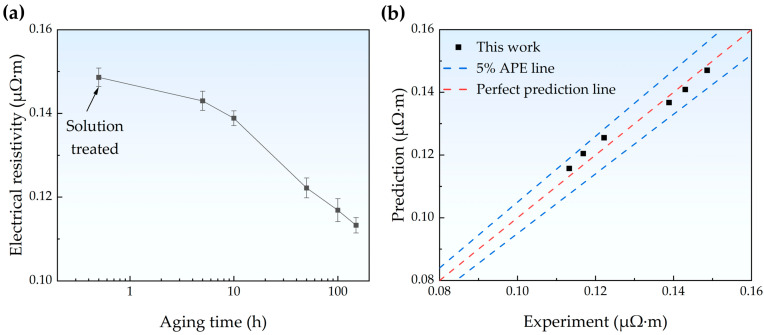
(**a**) Variation in electrical resistivity for the Fe-Cu alloy samples with aging time. (**b**) Comparison between the experimental and predicted electrical resistivity of the solution-treated and aged samples. The blue line represents the 5% absolute percentage error (APE) line, while the red line represents the perfect prediction line.

The electrical resistivity results measured through the four-point method are shown in Figure 9a. With increasing aging time, the resistivity of the Fe-Cu alloy gradually decreased from 0.1483 μΩ·m to 0.1132 μΩ·m. The reduction in resistivity is lower than that calculated according to Equation (1), which is similar to that reported in the literature [14]. In this section, we developed an electrical resistivity prediction model based on microstructural features. The quantitative microstructural information obtained through multiscale characterization using XRD, EBSD, and TEM provides data for the microstructure-based modeling.

According to Matthiessen’s rule, the electrical resistivity of the Fe-Cu alloy *ρ*_total_ can be decomposed into several contributions [52]:

(8) $$\rho_{\mathrm{total}}=\rho_{\mathrm{bulk}}+\rho_{D}+\rho_{\mathrm{GB}}+\rho_{\mathrm{CRP}}$$

where *ρ*_bulk_ is the bulk resistivity of the Fe-Cu alloy and can be calculated using Norbury’s empirical rule [22]. According to [52], *ρ*_0_ at room temperature is 0.0997 μΩ·m, and *ρ*_bulk_ is 0.1469 μΩ·m. *ρ*_D_, *ρ*_GB_, and *ρ*_CRP_ are the resistivity contributions of the dislocations, grain boundaries, and CRPs, respectively. According to the work of Basinski et al. [53], the resistivity increment attributed to dislocations can be expressed as follows:

(9) $$\rho_{D}=E\Lambda$$

where *E* is an empirical constant related to the scattering of electrons by dislocations ranging from 0.6 to 2.2 × 10^−18^ μΩ·m^3^ [53,54], and Λ is the dislocation density.

For grain boundaries, Braunovic et al. proposed the following relationship between resistivity change and average grain size [55]:

(10) $$\rho_{\mathrm{GB}}=A/d$$

where *A* is an empirical constant related to the scattering of electrons by grain boundaries ranging from 0.56 to 2.6 × 10^−8^ μΩ·m^2^ [54], and *d* is the average equivalent circular diameters of grains.

Finally, the resistivity contribution from CRPs is evaluated using the Kelekanjeri equation [56,57] combined with Norbury’s empirical rule [22]:

(11) $$\rho_{\mathrm{CRP}}=B\frac{V_{f}^{4/3}}{r^{2}}-CV_{f}$$

On the right side of the equation, the first term describes the scattering of electrons by the formation of CRPs, and the second term compensates for the effect of Cu atom precipitation on the bulk resistivity. Although the formation of CRPs contributes to additional resistivity, the reduction in solute atoms in the matrix leads to a decrease in resistivity, which is a competitive mechanism between the two.

In this work, E, A, B, and C are defined as optimizable parameters, with reasonable optimization ranges assigned according to their physical significance. An algorithm based on the minimize method was used to optimize these parameters, and the optimized values were E = 1.61 × 10^−18^ μΩ·m^3^, A = 1.10 × 10^−8^ μΩ·m^2^, B = 8.93 × 10^−17^ μΩ·m^3^, and C = 2.03 μΩ·m. The prediction performance of the model was characterized by the absolute percentage error (APE) and mean absolute percentage error (MAPE) between the prediction and experimental values, which can be calculated using the following formula:

(12) $$APE=\left|\frac{P_{i}-E_{i}}{E_{i}}\right|\times 100\%$$

(13) $$MAPE=\frac{1}{N}\sum_{i}\left|\frac{P_{i}-E_{i}}{E_{i}}\right|\times 100\%$$

where *N* represents the number of data points; *P_i_* and *E_i_* are the prediction and experiment values, respectively. The optimized model demonstrated a good predictive performance, with all absolute percentage errors below 5%. The maximum absolute percentage error was 3.08%, and the mean absolute percentage error was 2.01%. This model can accurately predict the resistivity of the Fe-Cu alloy from microstructure parameters with a maximum absolute percentage error not exceeding 5%.

To further validate the model’s universality, we collected electrical resistivity for an SA508 Gr.3 RPV steel neutron irradiated to 0.08dpa and applied the model for calculation [58]. During irradiation, microstructural changes other than solute-rich precipitates were neglected. The average radius and volume fraction of the solute-rich precipitates can be estimated to be 0.70 nm and 0.6% according to [59,60], since there are no characterization data of solute-rich precipitates in the aforementioned study [58]. The resistivity contribution from solute precipitates was found to be 0.186 μΩ·m, which was calculated using the precipitate part of the model, and the experimentally measured value of resistivity change was 0.593 μΩ·m, as reported in the literature [58]. Considering that the solute precipitates formed in the cited RPV steel for resistivity data are MNPs rather than CRPs, and the microstructure data were estimated from other experimental sets with similar irradiation conditions and material states, the discrepancy between the calculated results and the experimental results is acceptable. Specifically, the calculation results show that the resistivity of RPV steel increased after neutron irradiation, which is consistent with the observed phenomenon in the experiment, and the traditional alloy resistivity empirical law cannot explain this phenomenon. Meanwhile, the model currently does not consider the effect of matrix damage. In actual neutron-irradiated RPV steels, matrix damages also undeniably impact, especially at high doses [60]. Assuming that corresponding microstructure and resistivity studies can be conducted around RPV steels, fully considering various microstructural changes during irradiation, the model can be further extended to other irradiation defect scenarios, which is also a future direction of work.

## 5. Conclusions

The present work investigated the evolution of microstructure and electrical resistivity in an Fe-Cu RPV model alloy at 450 °C. The microstructural changes during the aging process were quantitatively analyzed by XRD, EBSD, and TEM. The formation and growth of CRPs agreed well with the JMA theory, and the change in electrical resistivity was well correlated with the microstructure evolution. Using detailed quantitative data analysis, we have developed a resistivity prediction model that considers microstructural mechanisms. This model can accurately predict the resistivity of the Fe-Cu alloy from microstructure parameters with a maximum absolute percentage error not exceeding 5%. The model can potentially extend to scenarios with other solute-rich precipitates. This work provides a research paradigm for establishing the relationship between non-destructive testing signals and microstructural features.

Through this model, microstructure can serve as a bridge between non-destructive testing and service performance, laying the foundation for building an in situ multidimensional RPV irradiation embrittlement evaluation method based on electrical resistivity, which is also the focus of our future work. Further research is needed to extend this model through a quantitative analysis of the microstructure and properties of neutron-irradiated RPV steels. As many reactors are nearing the end of their design life (typically 40 years), there is an increasing demand for reliable methods for the in situ assessment of the degradation of RPVs.

## Figures and Tables

**Table 1:** The chemical composition of the Fe-Cu model alloy.

Element (wt. %)	Fe	Cu	C	Si	S	P
Fe-Cu	Bal.	1.17	0.06	0.09	<0.01	<0.01

**Table 2:** The statistics of dislocation density and grain size.

Samples	Dislocation Density (10^13^ m^−2^)	Mean Diameter (μm)
Solution treated	1.19	36.9
Aged—5 h	1.22	39.5
Aged—10 h	1.00	38.2
Aged—50 h	1.09	32.6
Aged—100 h	1.08	35.7
Aged—150 h	0.90	33.1

**Table 3:** The mean radius, number density, and volume fraction of the CRPs in aged samples.

Aging Time (h)	Mean Radius (nm)	Number Density (10^22^ m^−3^)	Volume Fraction (%)
5	4.41	1.37	0.49
10	4.88	1.65	0.80
50	5.28	2.92	1.80
100	5.79	2.49	2.03
150	6.90	1.50	2.06

## Data Availability

The original contributions presented in this study are included in the article. Further inquiries can be directed to the corresponding author.
